# Adamantyl carboxamides and acetamides as potent human 11β-hydroxysteroid dehydrogenase type 1 inhibitors

**DOI:** 10.1016/j.bmc.2012.08.056

**Published:** 2012-11-01

**Authors:** Xiangdong Su, Heather A. Halem, Mark P. Thomas, Cecile Moutrille, Michael D. Culler, Nigel Vicker, Barry V.L. Potter

**Affiliations:** a Medicinal Chemistry, Department of Pharmacy and Pharmacology, University of Bath, Bath BA2 7AY, UK; b IPSEN, Biomeasure Inc., 27 Maple Street, Milford, MA 01757, USA

**Keywords:** Hydroxysteroid dehydrogenase, 11β-HSD1 inhibitor, Adamantyl amide, Diabetes, Structure–activity relationship

## Abstract

The modulation of 11β-HSD1 activity with selective inhibitors has beneficial effects on various metabolic disorders including insulin resistance, dyslipidemia and obesity. Here we report the discovery of a series of novel adamantyl carboxamide and acetamide derivatives as selective inhibitors of human 11β-HSD1 in HEK-293 cells transfected with the HSD11B1 gene. Optimization based on an initially identified 11β-HSD1 inhibitor (**3**) led to the discovery of potent inhibitors with IC_50_ values in the 100 nM range. These compounds are also highly selective 11β-HSD1 inhibitors with no activity against 11β-HSD2 and 17β-HSD1. Compound **15** (IC_50_ = 114 nM) with weak inhibitory activity against the key human cytochrome P450 enzymes and moderate stability in incubation with human liver microsomes is worthy of further development. Importantly, compound **41** (IC_50_ = 280 nM) provides a new lead that incorporates an adamantyl group surrogate and should enable further series diversification.

## Graphical abstract

Series of adamantyl carboxamide and acetamide derivatives were identified, providing potent and selective inhibitors of the therapeutic target human 11β-hydroxysteroid dehydrogenase type 1.

## 1 Introduction

**Scheme 1:**
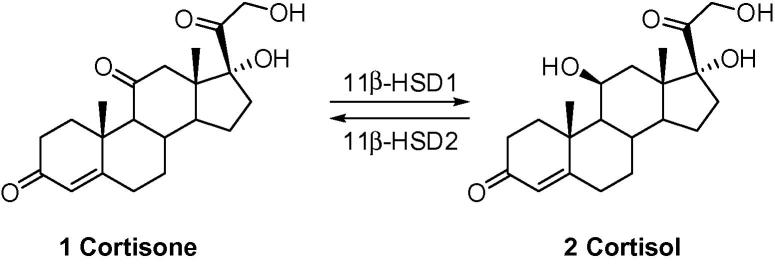
Interconversion of glucocorticoid catalyzed by 11β-HSDs.

11β-Hydroxysteroid dehydrogenase isozymes catalyze the intracellular conversion of inert 11-keto glucocorticoids to physiologically active glucocorticoids in specific tissues, and vice versa. 11β-Hydroxysteroid dehydrogenase type 1 (11β-HSD1) functions as an NADPH-dependent reductase converting cortisone (**1**) in humans to the active glucocorticoid cortisol (**2**) (Scheme 1). This endoplasmic reticulum-associated enzyme is highly expressed in liver, adipose tissue and in the central nervous system. 11β-HSD1 mediates the pre-receptor activation of cortisone, which provides a mechanism for specific tissues to produce intracellular, non-adrenal cortisol, thereby locally amplifying glucocorticoid action.^1–3^

Conversely, 11β-hydroxysteroid dehydrogenase type 2 (11β-HSD2) is an exclusively NAD^+^ dependent enzyme that catalyzes the transformation of cortisol to inactive cortisone. As the enzyme is highly expressed in mineralocorticoid target tissues, its glucocorticoid inactivation function prevents cortisol occupying the mineralocorticoid receptor (MR) which may lead to sodium retention, hypokalemia and hypertension.^4,5^

Excessive glucocorticoid action has been implicated in the development of several phenotypes associated with metabolic syndrome, such as obesity and type 2 diabetes.^6–8^ Studies show that glucocorticoid receptor (GR) activation stimulates hepatic glucose production, antagonises insulin secretion from pancreatic β-cells and insulin-mediated glucose uptake in peripheral tissues.^9^

The relationship of 11β-HSD1 mediated glucocorticoid regulation with metabolic disorders, such as obesity and type 2 diabetes has been well established by studies using genetically- modified rodent models. 11β-HSD1 knock-out mice resist diet-induced obesity and stress induced hyperglycaemia. These animals also have decreased cholesterol and triglyceride levels.^2,10,11^ In contrast, transgenic mice that overexpress 11β-HSD1 in fat tissues develop symptoms of insulin-resistant diabetes, hyperlipidemia and visceral obesity.^12,13^

The strategy of treating phenotypes of metabolic syndrome with selective 11β-HSD1 inhibitors has attracted considerable interest over the last decade. The discovery of various structural types of selective and potent 11β-HSD1 inhibitor has been extensively reviewed.^14–17^ Several crystal structures of 11β-HSD1 with different ligands have been published.^18^

Preclinical studies show that modulation of 11β-HSD1 activity with selective inhibitors has beneficial effects on various metabolic disorders including insulin resistance, dyslipidemia and obesity.^13,19–21^ During recent years, clinical studies have been conducted for several 11β-HSD1 inhibitors, including Pfizer’s PF-915275, Amgen’s AMG-221, AstraZeneca’s AZD-4017, Merck’s MK-0736, MK-0916 and Incyte’s INCB013739. The mid-stage study of INCB013739 demonstrated that treatment of type 2 diabetes mellitus patients with INCB013739 for 28 days significantly improved hepatic and peripheral insulin sensitivity and reduced fasting plasma glucose, plasma low density lipoprotein and total cholesterol levels.^22,23^

**Figure 1:**
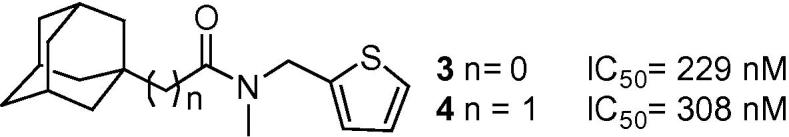
Adamantyl amides as 11β-HSD1 inhibitors.

To indentify novel 11β-HSD1 inhibitors, we have previously synthesized series of adamantyl ethanone compounds, and discovered several selective potent inhibitors.^24–26^ Our previous studies also identified compounds with a thiophene ring attached to an adamantyl moiety through an amide linker that possess high inhibitory activity against human 11β-HSD1. Compounds **3** and **4** (Fig. 1) exhibit inhibition of 11β-HSD1 with IC_50_ values in the 200-300 nM range when tested in the HEK-293 cell line transfected with the human 11β-HSD1 gene.^27^ We performed further optimization on these hit compounds by varying the aromatic region and linker. Target compounds were screened for their inhibitory activity against human 11β-HSD1 in a HEK293 cell based assay.^28^ Potent 11β-HSD1 inhibitors were also examined for their selectivity over human 11β-HSD2 and 17β-hydroxysteroid dehydrogenase type 1 (17β-HSD1), one of the alcohol oxidoreductases that catalyze the NADPH-dependent reduction of estrone to estradiol.^29^ Here we report the synthesis and the structure–activity relationships of some adamantyl carboxamide and adamantyl acetamide compounds as potent inhibitors of 11β-HSD1.

## 2 Results and discussion

### 2.1 Synthesis of target compounds

**Scheme 2:**
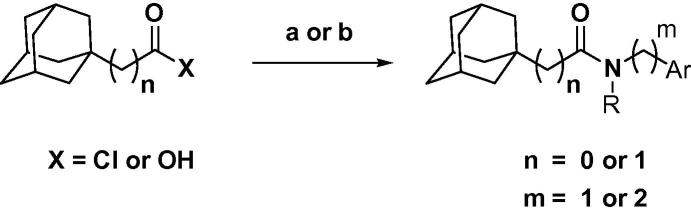
Synthesis of adamantyl carboxamides and acetamides. Reagents and conditions: (a) X = Cl, amine, Et_3_N/DCM, rt; (b) X = OH, amine, EDCI, DMAP, Et_3_N/DCM, rt.

Target compounds with an adamantyl group were synthesized from 1-adamantyl carbonyl chloride or 1-adamantyl acetic acid through an amide coupling reaction with various amines under standard conditions as illustrated in Scheme 2. Amines were either purchased or made by reductive amination from the corresponding aldehyde derivatives.

### 2.2 Structure–activity relationship

11β-HSD1 inhibition was measured in intact human HEK-293 cells to reveal the true inhibitory activity. Differences in observed effects for different compounds may reflect combinatorial factors including the ability to penetrate the cell membrane, the stability within the cell and the binding ability to the enzyme.

Our previous study found a pharmacophore template that consists of a thiophenyl ring attached to an adamantyl moiety through an amide linker. The adamantyl group, being highly hydrophobic, has been identified as a popular moiety in different structural combinations for 11β-HSD1 inhibition.^30–32^ Compounds **3** and **4** (Fig. 1) exhibit inhibition of 11β-HSD1 with IC_50_ values in the 200–300 nM range. Further optimization was performed based on these compounds.

The substituent effects on the thiophenyl ring were evaluated with compounds **6**–**11** Table 1. The 3-methyl derivative **6** retains nearly the same activity as parent compound **3**. However, the amino group substitution at the same position (**7**) causes a sharp decrease of activity, suggesting that a basic and hydrophilic substitution is not favoured in that area. Moving the methyl substituent to the 4- or 5-position affords compounds **8** or **10**, respectively. These compounds show only weak inhibition for 11β-HSD1 at a concentration of 1 μM, as does the compound with 5-chloro substituent (**11**). Compared with the 2-thiophenyl compound (**3**), the 3-thiophenyl analogue (**12**) exhibits an increased activity with an IC_50_ value of 125 nM.^27^

The effect of using another 5-membered heterocycle to replace the thiophenyl ring was investigated Table 1. The 2-furanyl derivative (**13**) with an IC_50_ at 249 nM has about the same potency as **3.**^27^ The 1-methyl-1*H*-2-pyrrolyl compound (**15**) with an IC_50_ of 114 nM exhibits a twofold higher potency than **3**. It is assumed that the pyrrolyl ring can form further interactions with the enzyme and/or alters the geometry of the molecule placing the adamantyl and/or the aromatic ring in a position to gain further binding in the active site. Replacing the thiophenyl group with a 1-methyl-1*H*-imidazolyl, 1-methyl-1*H*-pyrazolyl or 4-methyl-thiazolyl group generated compounds **17**–**19**. None of these compounds shows significant inhibitory activity at 1 μM.

It was found that the *N*-methyl amide analogue usually exhibits higher potency than the corresponding NH analogues; for example, compound **3** is almost twice as active as **5**, while compound **15** is over 15 times more potent than **16**.

In the six-membered aromatic ring series, the 3-amino substituted benzene derivative (**20**) exhibits an IC_50_ value of 118 nM, suggesting that the NH_2_ group may enable more favourable interactions with the enzyme. On the other hand, the acetamido-, methyl- or chloro- substitution at the same position (**21**–**23**) fails to generate a compound with significant inhibitory activity. The pyridyl derivatives **24**–**27** only exhibit poor to modest activity Table 2.

Compounds with a more flexible tethering were also synthesized and evaluated for their activity. (Table 3). The thiophenyl derivative (**28**) has only half the potency of compound **3.** However, the 2-pyridyl compound (**30**) exhibits a significant inhibition with an IC_50_ value of 165 nM, suggesting that the flexible linker is more suitable for a pyridyl derivative.

In the adamantyl acetamide series, the 2-thiophenyl compound (**4**) is only slightly weaker than the corresponding adamantyl amide (**3**), while the 3-thiophenyl derivative **34** exhibits a greater than 2-fold reduction in potency compared to **12**.^27^ Furthermore, the 2-pyrrolyl derivative (**35**) with an IC_50_ of 655 nM loses nearly 6-fold inhibitory activity compared with 2-pyrrolyl compound **15** (IC_50_ = 114 nM) in the amide series. It is also observed in the acetamide series that the *N*-methyl compounds **4** and **35** possess relatively higher potency than their respective NH analogues **33** and **36** Table 4.

Therefore, compound **15** is an interesting lead for further optimization and development in this class of inhibitors.

We have previous discovered that a substituted phenyl group may potentially be used as a replacement for the adamantyl group.^33^ We therefore pursued the synthesis of a set of polycyclic or substituted aliphatic cyclic compounds in the carboxamide series Table 5. The noradamantyl derivative (**39**) with an IC_50_ of 1080 nM was nearly fivefold less active than the adamantyl analogue **3**, although these two compounds have very similar molecular shape and properties. The (*p*-tolyl)cyclopropyl compound (**41**, IC_50_ = 280 nM) exhibits 11β-HSD1 inhibitory activity at the same potency as compound **3**, suggesting the possibility to be used as a replacement for adamantyl group. It is interesting to note that 4-chlorophenylcyclopropyl compound (**42**) shows a loss of activity of ∼5.5-fold with an IC_50_ of only ∼1.6 μM, in comparison with **41**; however, cyclobutyl analogue (**43**) achieves an improved activity with an IC_50_ value at 385 nM.

All potent compounds were evaluated for their selectivity over 11β-HSD2 and 17β-HSD1 enzymes. They exhibit no inhibitory activity at 10 μM.

To identify potential problems with these compounds interfering with the metabolism of other drugs, compounds **3**, **4, 15** and **41** were studied for their inhibition of the key human cytochrome P450 enzymes: 1A2, 2C9, 2C19, 2D6, 3A4-BFC (7-benzyloxy-4-(trifluoromethyl)coumarin) and 3A4-BQ (7-benzyloxyquinoline) (Table 6). Compound **3** displays weak activity against 2C9 and 3A4-BFC, while the acetamide analogue (**4**) strongly inhibits 3A4-BFC (IC_50_ = 150 nM). Nevertheless, compound **15**, one of the most potent 11β-HSD1 inhibitors in this series, exhibits very weak inhibition for these cytochrome P450 enzymes. The non-adamantyl compound **41** shows a moderate inhibition of 2CA, 3A4-BFC and 3A4-BQ with IC_50_ values from 1.6 to 7.3 μM.

Potent inhibitors (**3** and **15**) were incubated at 37 °C for 40 min with human liver microsomes in the presence of the cofactor NADPH to evaluate their stability under such conditions. The remaining parent compound was measured using a HPLC method. Both **3** and **15** are moderately stable under such condition with a *T*_1/2_ value from 60 to 70 min.

Compound **3** was studied for its membrane permeability using the Caco-2 cell model. The apparent permeability coefficient (*P*_app_) from apical to basolateral side was measured at a concentration of 20 μM; and the result shows that the compound is of high permeability (P_app_ (A>B) = 3.3 × 10^−5^).

As the inhibitory activity was studied on the whole cell, the high permeability of the inhibitor suggests a potentially good correlation between the activity and the binding affinity to the enzyme. The GOLD docking program was used to determine possible binding poses.

**Figure 2:**
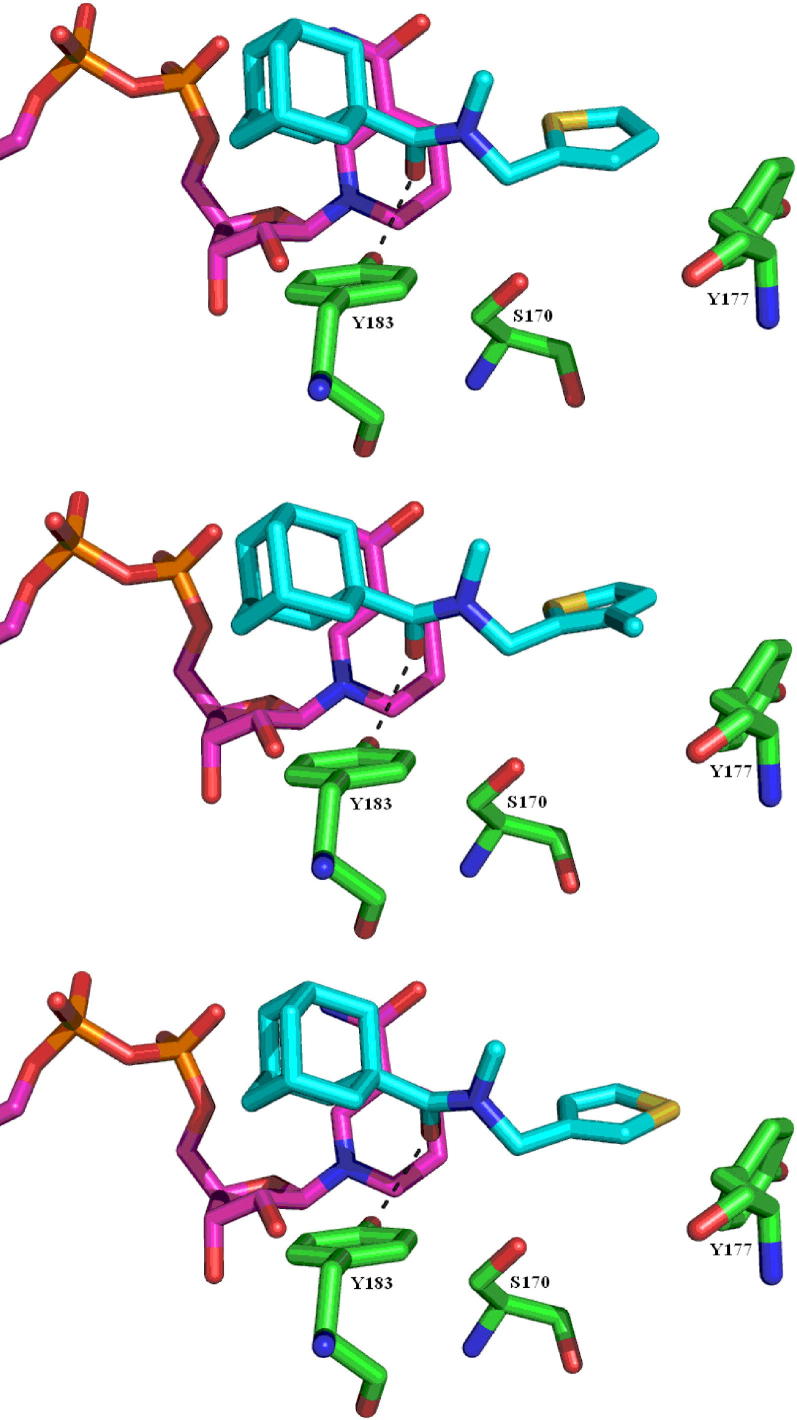
Docking solutions for compounds **3** (top), **6** (middle) and **12** (bottom), all in cyan, with the cofactor in pink and the active site residues in green.

The top 5 docking solutions for compound **3** are almost identical. The adamantyl group is positioned in a hydrophobic pocket close to the nicotinamide ring of the co-factor, the same orientation as in the published X-ray crystal structure. The carbonyl group, being 2.5 Å away from the catalytic residue Tyr183, may form a hydrogen bond interaction with the residue. The thiophene ring is about 3.6 Å from Tyr177, suggesting an edge-on interaction (Fig. 2, top). The best 5 docking solutions for compound **6** are all adapted to the same conformation as that of compound **3**, with the extra methyl group being closer to the residue Tyr177 (2.6 Å) (Figure 2, middle). The top 5 poses of compound **12** also overlap perfectly with that of compound **3**, with the exception of the sulfur atom pointing towards the hydroxyl group of Tyr177 (3 Å), suggesting a further hydrogen bond interaction (Figure 2, bottom).

**Figure 3:**
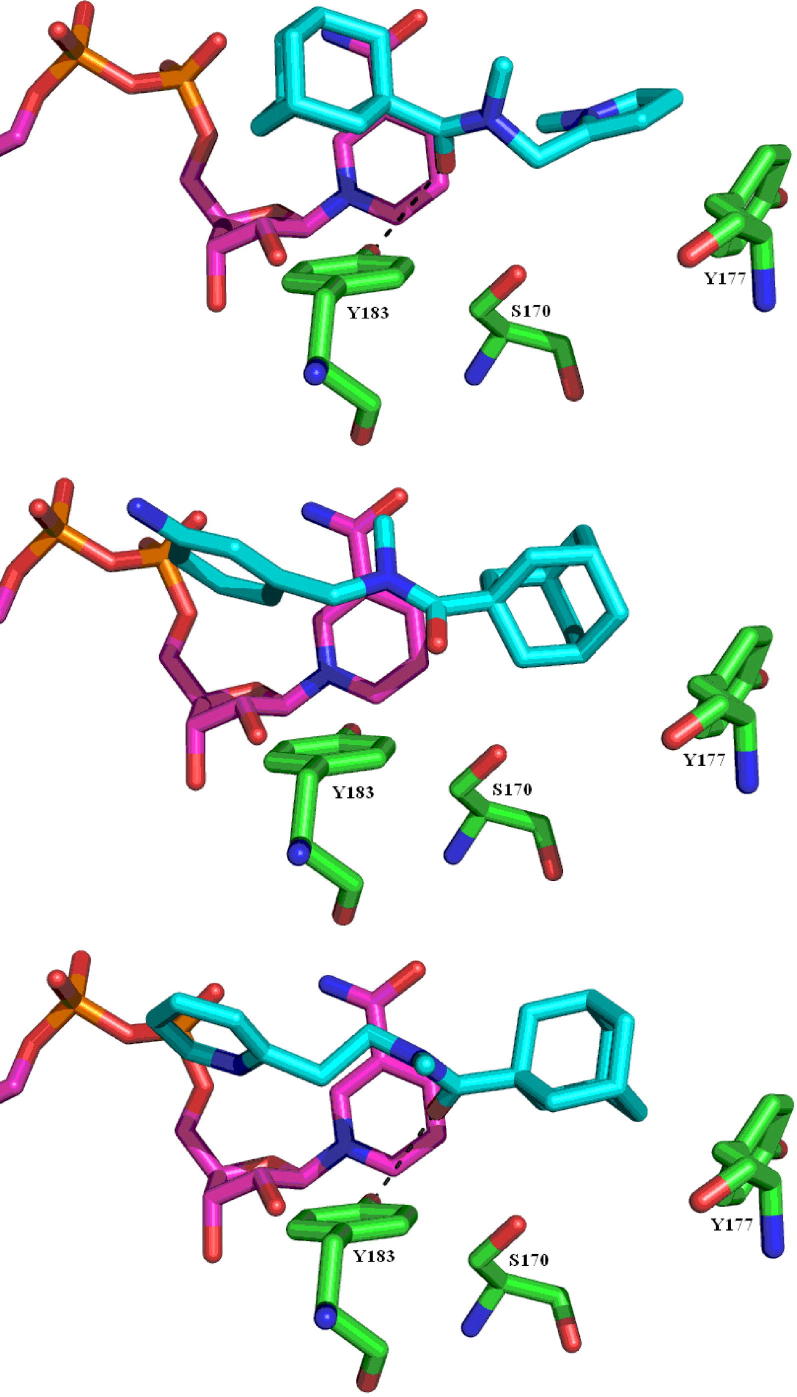
Docking solutions for compounds **15** (top), **20** (middle) and **30** (bottom), all in cyan, with the cofactor in pink and the active site residues in green.

The top docked conformation of compound **15** is very similar to that of **3** with the adamantyl group close to the NADP. The oxygen of the amide carbonyl group is positioned to form hydrogen bond interactions with Tyr183 (2.7 Å) and Ser 170 (3 Å). The pyrrole ring, being ∼3.5 Å from Tyr177 may form a stacking interaction with the residue (Fig. 3, top). On the other hand, docking studies of compound **20** show that the top 5 poses position the phenyl ring in the hydrophobic pocket close to the cofactor. The amino group, being 2.9 Å away from Thr124, possibly forms added hydrogen bond interactions. With the ‘reversed’ orientation, the adamantyl group fits into the pocket formed by Ser170, Ala172, Tyr177 and Val180 (Figure 3, middle). The best docking solutions for compound **30** also adapted to the ‘reversed’ position with the adamantyl group forming edge-on interactions with Tyr177 (3.7 Å), and the carbonyl group may form hydrogen bond interactions with the catalytic residue Tyr183 (2.8 Å) and Ser170 (3.4 Å) (Fig. 3, bottom).

## 3 Conclusion

Series of adamantyl carboxamide and acetamide derivatives were synthesized and their ability to inhibit human 11β-HSD1 was evaluated on a HEK-293 cell line stably transfected with the HSD11B1 gene. Based on the initially identified 11β-HSD1 inhibitor (**3**) further optimization in the aromatic region, the carbon tethering and adamantyl group in the molecule led to the discovery of potent inhibitors with an IC_50_ value in the 100 nM range. The potent compounds are also highly selective 11β-HSD1 inhibitors with no activity against 11β-HSD2 and 17β-HSD1. Compound **15** exhibits very weak inhibitory activity against the key human cytochrome P450 enzymes, indicating that it is unlikely for the compound to interfere with metabolism of other drugs. In addition, the compound is also relatively stable when incubated with human liver microsomes. Compound **41** also offers a potent lead for further development with a (*p*-tolyl)cyclopropyl surrogate for the adamantyl group.

## 4 Experimental

### 4.1 11β-HSD1 inhibition assay protocol: human HEK-293 cell based assay

The cell-based assays were conducted on the human HEK-293 cell line stably transfected with the HSD11B1 gene.^28^ 11β-HSD1 activity was determined by measuring the amount of tritiated product formed using a scintillation proximity assay (SPA).

Cells were incubated in 96-well micro-plates in the presence of tritiated cortisone substrate and the assay plates contained internal high and low controls to allow calculation of percentage inhibition. Each well of a 96-well culture plate was seeded with human 11β-HSD1 overexpressed HEK293 cells in 100 μL medium. When the cells were 80% confluent, the medium was removed from each well then 100 μL of fresh, serum-free, medium containing ^3^H-cortisone (22 nM) and test compound in 1% DMSO was added. The control wells were also dispensed. The high control wells did not contain compound, while low controls did not contain cells. The plate was incubated at 37 °C for 2 h, after which 50 μL of medium was removed from each well and transferred to a microplate containing 100 μL of a pre-incubated mixture of anti-cortisol antibody and SPA bead. The mixture was incubated with gentle shaking until equilibrium was reached, before transferring to a scintillation counter to establish the enzyme activity in each sample.

The compounds were initially tested at a concentration of 1 μM. Those compounds showing over 70% inhibition of 11β-HSD1 at 1 μM were subjected to an IC_50_ measurement. A Merck compound 544 was used as the reference compound in the assay (IC_50_ = 50 ± 20 nM).^19^ The IC_50_ values are reported as the mean value of 3 measurements with variance less than 20%. IC_50_ values were calculated from the dose-response curve using Microsoft Excel add-in XLfit with a Four Parameter Logistic nonlinear regression model.

### 4.2 11β-HSD2 and 17β-HSD1 inhibition assay protocol

11β-HSD2 activity was measured in whole Chinese hamster ovary (CHO) cells stably transfected with the HSD11B2 gene. Cells were incubated in 96-well microplates in the presence of tritiated cortisol (22nM) with or without the inhibitor to be tested. After incubation at 37 °C for 2 h, the mixture was extracted with ethyl acetate. Enzyme activity was determined by measuring the amount of tritiated product using thin layer chromatography (Chloroform/Ethanol 92:8 v/v) with a PhosphorImager.

17β-HSD1 inhibition assay was performed according to the literature procedure.^29^

### 4.3 Molecular modelling

Selected ligands were docked into the human 11β-HSD1 protein X-ray crystal structure 2ILT^34^ using the GOLD docking program in version 5.1 of the GOLD suite with default settings in the presence of the cofactor. The binding site was defined as a sphere of 10 Å radius around the centroid of the ligand in the 2ILT structure. Each ligand was docked 25 times. The Goldscore scoring function was used to rank the ligands in order of fitness to generate the best poses of the ligand in the active site. The five most highly ranked poses of each ligand were studied.

### 4.4 Chemistry

All chemicals were purchased from either Aldrich Chemical Co. (Gillingham, UK) or Alfa Aesar (Heysham, UK). All organic solvents of AR grade were supplied by Fisher Scientific (Loughborough, UK). Compounds **3**–**5, 12, 13, 28, 29** were synthesized according to literature procedures.^27^ Melting points were determined using a Stanford Research Systems Optimelt MPA100 and are uncorrected. Compounds in solid form were crystallized from dichloromethane (DCM)-ethyl acetate solution. Thin layer chromatography (TLC) was performed on pre-coated aluminum plates (Merck, silica gel 60 F_254_). Products were visualized either by UV irradiation at 254 nm and by staining with 5% w/v molybdophosphoric acid in ethanol, followed by heating. Flash column chromatography was performed on pre-packed columns (RediSep Rf) and gradient elution (solvents indicated in text) on the Combiflash RF system (Teledyne Isco). ^1^H NMR spectra were recorded with a Jeol Delta 270 MHz spectrometer. Chemical shifts are reported in parts per million (ppm, *δ*) relative to tetramethylsilane (TMS) as an internal standard. LC/MS spectra were performed on a Waters 2790 machine with Waters ‘Symmetry’ C18 column (packing: 3.5 μm, 4.6 × 75 mm) eluting with 10% H_2_O/CH_3_OH (1 mL/min), and detected with a ZQ MicroMass spectrometer and PDA detector using Atmospheric Pressure Chemical Ionization (APCI) or Electrospray Ionisation (ESI). High resolution mass spectra were recorded on a Bruker MicroTOF with ESI. HPLC was undertaken using a Waters 717 machine with Autosampler and PDA detector. The column used was a Waters ‘Symmetry’ C18 (packing: 3.5 μm, 4.6 × 150 mm) with an isocratic mobile phase consisting of H_2_O/CH_3_CN at a flow rate of 1.0 mL/min.

#### 4.4.1 General method A for the preparation of adamantyl carboxamide derivatives **6**–**32**

To a solution of the 1-adamantane carbonyl chloride (0.50 mmol, 1.06 equiv) in DCM (5 mL) was added triethylamine (0.15 mL), followed by the corresponding amine (0.47 mmol, 1 equiv). The reaction mixture was stirred at ambient temperature under nitrogen overnight. PS-Trisamine (10-20 mg) was added. After stirring at ambient temperature for another 2 h, the mixture was filtered and evaporation of the solvent gave a residue that was purified by flash chromatography (ethyl acetate-DCM gradient elution) to give the desired carboxamide derivatives.

#### 4.4.2 General method B for the preparation of acetamide or carboxamide derivatives **33**–**44**

To a solution of the corresponding carboxylic acid (0.5 mmol) in DCM (8 mL) were added 1-ethyl-3-(3-dimethylaminopropyl)-carbodiimide (EDCI, 0.6 mmol), 4-(dimethylamino) pyridine (DMAP, 10 mg) and triethylamine (0.1 mL) at room temperature. After stirring for 0.5 h, the corresponding amine (0.5 mmol) was added to the reaction mixture. After 12 h the mixture was partitioned between DCM and brine and the organic layer was washed with brine, dried over MgSO_4_ and concentrated in vacuo*.* The crude product was purified with flash chromatography (Ethyl acetate-DCM gradient elution) to give the target acetamide or carboxamide.

#### 4.4.3 *N*-Methyl-*N*-((3-methylthiophen-2-yl)methyl)adamantane-1-carboxamide (**6**)

A white solid was obtained in 92% yield. mp 72–73.5 °C; ^1^H NMR (CDCl_3_) *δ* 1.71 (6H, s), 2.04 (9H, s), 2.20 (3H, s), 3.05 (3H, s), 4.68 (2H, s), 6.77 (1H, d, *J* = 5.2 Hz) and 7.10 (1H, d, *J* = 5.2 Hz); LC/MS (APCI) *m*/*z* 304 (M+H)^+^; HRMS (ESI) calcd for C_18_H_26_NOS (M+H)^+^ 304.1735, found: 304.1732; HPLC *t*_R_ 8.1 min (>99%).

#### 4.4.4 *N*-((3-Aminothiophen-2-yl)methyl)-*N*-methyladamantane-1-carboxamide (**7**)

A light yellow solid was obtained in 77% yield. mp 117–119 °C; ^1^H NMR (CDCl_3_) *δ* 1.70 (6H, s), 2.01 (9H, s), 3.21 (3H, s), 4.34 (2H, br, NH_2_), 4.47 (2H, s), 6.54 (1H, d, *J* = 5.3 Hz) and 6.97 (1H, d, *J* = 5.5 H); LC/MS (APCI) *m*/*z* 303 (M−H)^+^; HRMS (ESI) calcd for C_17_H_25_N_2_OS (M+H)^+^ 305.1688, found: 305.1685; HPLC *t*_R_ 2.6 min (>98%).

#### 4.4.5 *N*-Methyl-*N*-((4-methylthiophen-2-yl)-methyl)adamantane-1-carboxamide (**8**)

A white solid was obtained in 82% yield. mp 87–88 °C; ^1^H NMR (CDCl_3_) *δ* 1.70 (6H, S), 2.03 (9H, s), 2.20 (3H, d, *J* = 1.0 Hz), 3.06 (3H, s), 4.67 (2H, s), 6.71 (1H, s) and 6.77 (1H, q, *J* = 1.2 Hz); LC/MS (APCI) *m*/*z* 304 (M+H)^+^; HRMS (ESI) calcd for C_18_H_26_NOS (M+H)^+^ 304.1735, found: 304.1738; HPLC *t*_R_ 8.3 min (>99%).

#### 4.4.6 *N*-((4-Methylthiophen-2-yl)methyl)-adamantane-1-carboxamide (**9**)

A white solid was obtained in 95% yield. mp 127–128.5 °C; ^1^H NMR (CDCl_3_) *δ* 1.65–1.91 (12H, m), 2.03 (3H, br), 2.20 (3H, d, *J* = 1.0 Hz), 4.53 (2H, d, *J* = 5.4 Hz_2_), 5.86 (1H, br, NH) and 6.74–6.77 (2H, m); LC/MS (APCI) *m*/*z* 288 (M−H)^+^; HRMS (ESI) calcd for C_17_H_24_NOS (M+H)^+^ 290.1579, found: 290.1583; HPLC *t*_R_ 5.5 min (>99%).

#### 4.4.7 *N*-Methyl-*N*-((5-methylthiophen-2-yl)-methyl)adamantane-1-carboxamide (**10**)

A white solid was obtained in 73% yield. mp 83–85.5 °C; ^1^H NMR (CDCl_3_) *δ* 1.70 (6H, s), 2.03 (9H, s, 2.42 (3H, d, *J* = 1.0 Hz), 3.05 (3H, s), 4.64 (2H, s), 6.55 (1H, dq, *J* = 3.3, 1.0 Hz) and 6.68 (1H, d, *J* = 3.3 Hz); LC/MS (APCI) *m*/*z* 304 (M+H)^+^; HRMS (ESI) calcd for C_18_H_26_NOS (M+H)^+^ 304.1735, found: 304.1731; HPLC *t*_R_ 7.0 min (99%).

#### 4.4.8 *N*-((5-Chlorothiophen-2-yl)methyl)-*N*-methyladamantane-1-carboxamide (**11**)

A white solid was obtained in 74% yield. mp 92–94 °C; ^1^H NMR (CDCl_3_) *δ* 1.75 (6H, s), 2.01 (9H, s), 3.10 (3H, s), 4.55 (2H, s) and 6.70 (2H, AB); LC/MS (APCI) *m*/*z* 324 (M+H)^+^; HRMS (ESI) calcd for C_17_H_23_ClNOS (M+H)^+^ 324.1189, found: 324.1193; HPLC *t*_R_ 10.0 min (99%).

#### 4.4.9 *N*-(Furan-2-ylmethyl)adamantane-1-carboxamide (**14**)

A white solid was obtained in 78% yield. mp 69–72 °C; ^1^H NMR (CDCl_3_) *δ* 1.65–1.75 (6H, m), 1.85 (6H, d, *J* = 2.8 Hz), 2.03 (3H, br), 4.41 (2H, d, *J* = 5.8 Hz), 5.85 (1H, br, NH), 6.19 (1H, d, *J* = 2.8 Hz), 6.30 (1H, dd, *J* = 3.3, 2.0 Hz) and 7.34 (1H,d, *J* = 1.9 Hz); LC/MS (APCI) *m*/*z* 258 (M-H)^+^; HRMS (ESI) calcd for C_16_H_22_NO_2_ (M+H)^+^ 260.1651, found: 260.1667; HPLC *t*_R_ 2.6 min (99%).

#### 4.4.10 *N*-Methyl-*N*-((1-methyl-1*H*-pyrrol-2-yl)-methyl)adamantane-1-carboxamide (**15**)

A white solid was obtained in 87% yield. mp 104–105.5 °C; ^1^H NMR (CDCl_3_) *δ* 1.76 (6H, s), 2.02 (9H, s), 3.02 (3H, s), 3.50 (3H, s), 4.61 (2H, s), 6.03 (2H, d, *J* = 2.3 Hz) and 6.58 (1H, t, *J* = 2.2 Hz); LC/MS (APCI) *m*/*z* 287 (M+H)^+^; HRMS (ESI) calcd for C_18_H_27_N_2_O (M+H)^+^ 287.2123, found: 287.2108; HPLC *t*_R_ 6.6 min (99%).

#### 4.4.11 *N*-((1-Methyl-1*H*-pyrrol-2-yl)meth-yl)-adamantane-1-carboxamide (**16**)

A white solid was obtained in 76% yield. mp 117–119 °C; ^1^H NMR (CDCl_3_) *δ* 1.58–1.75 (6H, m), 1.83 (6H, d, *J* = 2.8 Hz), 2.02 (3H, br s), 3.53 (3H, s), 4.42 (2H, d, *J* = 5.2 Hz), 5.60 (1H, br, NH), 6.05 (2H, d, *J* = 2.2 Hz) and 6.65 (1H,t, *J* = 2.2 Hz); LC/MS (APCI) *m*/*z* 271 (M−H)^+^; HRMS (ESI) calcd for C_17_H_24_N_2_NaO (M+Na)^+^ 295.1786, found 295.1768; HPLC *t*_R_ 2.7 min (99%).

#### 4.4.12 *N*-Methyl-*N*-((1-methyl-1*H*-imidazol-2-yl)methyl)adamantane-1-carboxamide (**17**)

A white solid was obtained in 50% yield. mp 175–176 °C; ^1^H NMR (CDCl_3_) *δ* 1.65–1.83 (6H, m), 1.98 (6H, d, *J* = 2.7 Hz), 2.03 (3H, br s), 3.33 (3H, s), 3.76 (3H, s), 4.82 (2H, s), 6.88 (1H, d, *J* = 1.9 Hz) and 7.14 (1H, d, *J* = 1.9 Hz); LC/MS (ESI) *m*/*z* 310 (M+Na)^+^; HRMS (ESI) calcd for C_17_H_26_N_3_O (M+H)^+^ 288.2076, found 288.2079; HPLC *t*_R_ 6.6 min (99%).

#### 4.4.13 *N*-Methyl-*N*-((1-methyl-1*H*-pyrazol-5-yl)-methyl)adamantane-1-carboxamide (**18**)

A white solid was obtained in 90% yield. mp 102–103.5 °C; ^1^H NMR (CDCl_3_) *δ* 1.71 (6H, s), 2.01 (9H, s), 3.10 (3H, s), 3.70 (3H, s), 4.58 (2H, s) and 5.87 (1H, s); LC/MS (ESI) *m*/*z* 288 (M+H)^+^; HRMS (ESI) calcd for C_17_H_26_N_3_O (M+H)^+^ 288.2076, found: 288.2068; HPLC *t*_R_ 5.3 min (99%).

#### 4.4.14 *N*-Methyl-*N*-((4-methylthiazol-2-yl)-methyl)adamantane-1-carboxamide (**19**)

A colourless thick oil was obtained in 88% yield. ^1^H NMR (CDCl_3_) *δ* 1.71 (6H, s), 2.03 (9H, s), 2.41 (3H, s), 3.19 (3H, s), 4.80 (2H, s) and 6.81 (1H, s); LC/MS (ESI) *m*/*z* 305 (M+H)^+^; HRMS (ESI) calcd for C_17_H_25_N_2_OS (M+H)^+^ 305.1688, found: 305.1672; HPLC *t*_R_ 6.0 min (99%).

#### 4.4.15 *N*-(3-Aminobenzyl)-*N*-methyladam-antane-1-carboxamide (**20**)

An off-white solid was obtained in 88% yield. mp 105–106.5 °C; ^1^H NMR (CDCl_3_) *δ* 1.70 (6H, s), 2.04 (9H, s), 2.94 (3H, s), 3.65 (2H, s, NH_2_), 4.60 (2H, s), 6.49 (1H, br), 6.54-6.59 (2H, m) and 7.09 (1H, t, *J* = 7.6 Hz); LC/MS (ESI) *m*/*z* 299 (M+H)^+^; HRMS (ESI) calcd for C_19_H_27_N_2_O (M+H)^+^ 299.2123, found: 299.2117; HPLC *t*_R_ 2.3 min (99%).

#### 4.4.16 *N*-(3-Acetamidobenzyl)-*N*-methyl-adamantane-1-carboxamide (**21**)

A white solid was obtained in 55% yield. mp 109–111 °C; ^1^H NMR (CDCl_3_) *δ* 1.70 (6H, s), 2.03 (9H, s), 2.16 (3H, s), 2.95 (3H, s), 4.63 (2H, s_2_), 6.89 (1H, br), 7.12 (2H, m), 7.45 (1H, t, *J* = 8.7 Hz) and 7.51 (1H, s, NH); LC/MS (ESI) *m*/*z* 339 (M−H)^+^; HRMS (ESI) calcd for C_21_H_29_N_2_O_2_ (M+H)^+^ 341.2229, found: 341.2211; HPLC *t*_R_ 4.8 min (99%).

#### 4.4.17 *N*-Methyl-*N*-(4-methylbenzyl)adam-antane-1-carboxamide (**22**)

A white solid was obtained in 87% yield. mp 93–95 °C; ^1^H NMR (CDCl_3_) *δ* 1.70 (6H, s, 3 × CH_2_), 2.04 (9H, s), 2.32 (3H, s), 2.93 (3H, s), 4.65 (2H, s) and 7.10 (4H, AB); LC/MS (ESI) *m*/*z* 298 (M+H)^+^; HRMS (ESI) calcd for C_20_H_28_NO (M+H)^+^ 298.2171, found: 298.2189; HPLC *t*_R_ 5.9 min (99%).

#### 4.4.18 *N*-(4-Chlorobenzyl)-*N*-methyladam-antane-1-carboxamide (**23**)

A white solid was obtained in 54% yield. mp 65–66.5 °C; ^1^H NMR (CDCl_3_) *δ* 1.71 (6H, s), 2.03 (9H, s), 2.98 (3H, s_3_), 4.61 (2H, s) and 7.20 (4H, AB); LC/MS (ESI) *m*/*z* 318 (M+H)^+^; HRMS (ESI) calcd for C_19_H_25_ClNO (M+H)^+^ 318.1625, found: 318.1615; HPLC *t*_R_ 6.2 min (99%).

#### 4.4.19 *N*-Methyl-*N*-(pyridin-3-ylmethyl)-adamantane-1-carboxamide (**24**)

A white solid was obtained in 91% yield. mp 57–59.5 °C; ^1^H NMR (CDCl_3_) *δ* 1.70 (6H, s), 2.03 (9H, s), 3.05 (3H, s), 4.62 (2H, s), 7.25 (1H, dd, *J* = 7.9, 5.0 Hz), 7.53 (1H, dt, *J* = 7.9, 1.7 Hz), 8.46 (1H, d, *J* = 1.7 Hz) and 8.50 (1H, dd, *J* = 4.9, 1.5 Hz); LC/MS (ESI) *m*/*z* 285 (M+H)^+^; HRMS (ESI) calcd for C_18_H_25_N_2_O (M+H)^+^ 285.1967, found: 285.1963; HPLC *t*_R_ 5.5 min (99%).

#### 4.4.20 *N*-(Pyridin-3-ylmethyl)adamantane-1-carboxamide (**25**)

A white solid was obtained in 76% yield. mp 79–81.5 °C; ^1^H NMR (CDCl_3_) *δ* 1.64–1.78 (6H, m), 1.86 (6H, d, *J* = 2.8 Hz), 2.04 (3H, br s), 4.44 (2H, d, *J* = 6.0 Hz), 5.94 (1H, br, NH), 7.24 (1H, m), 7.58 (1H, dt, *J* = 8.0, 1.7 Hz) and 8.52 (2H, m); LC/MS (ESI) *m*/*z* 269 (M−H)^+^; HRMS (ESI) calcd for C_17_H_23_N_2_O (M+H)^+^ 271.1810, found: 271.1816; HPLC *t*_R_ 2,3 min (99%).

#### 4.4.21 *N*-(Pyridin-2-ylmethyl)adamantane-1-carboxamide (**26**)

A white solid was obtained in 70% yield. mp 91–93.5 °C; ^1^H NMR (CDCl_3_) *δ* 1.70 (6H, m), 1.91 (6H, d, *J* = 3.0 Hz), 2.05 (3H, br s), 4.52 (2H, d, *J* = 4.7 Hz), 6.95 (1H, br, NH), 7.15–7.24 (2H, m), 7.64 (1H, td, *J* = 7.9, 2.0 Hz) and 8.53 (1H, d, *J* = 4.5 Hz); LC/MS (ESI) *m*/*z* 269 (M-H)^+^; HRMS (ESI) calcd for C_17_H_23_N_2_O (M+H)^+^ 271.1810, found: 271.1813; HPLC *t*_R_ 2,5 min (99%).

#### 4.4.22 *N*-Methyl-*N*-((6-methylpyridin-2-yl)-methyl)adamantane-1-carboxamide (**27**)

A white solid was obtained in 80% yield. mp 76–77.5 °C; ^1^H NMR (CDCl_3_) *δ* 1.69 (6H, s), 2.03 (9H, s), 2.53 (3H, s), 3.06 (3H, s), 4.76 (2H, s), 6.91 (1H, d, *J* = 7.6 Hz), 7.02 (1H, d, *J* = 7.6 Hz) and 7.53 (1H, t, *J* = 7.7 Hz); LC/MS (ESI) *m*/*z m*/*z* 299 (M+H)^+^; HRMS (ESI) calcd for C_19_H_27_N_2_O (M+H)^+^ 299.2123, found: 299.2121; HPLC *t*_R_ 6.0 min (99%).

#### 4.4.23 *N*-Methyl-*N*-(2-(pyridin-2-yl)ethyl)-adamantane-1-carboxamide (**30**)

A white solid was obtained in 83% yield. mp 64–65 °C; ^1^H NMR (CDCl_3_) *δ* 1.69 (6H, s), 1.91–2.01 (9H, m), 3.01 (2H, t, *J* = 7.3 Hz), 3.04 (3H, s, NCH_3_), 3.73 (2H, t, *J* = 7.1 Hz), 7.10–7.25 (2H, m), 7.59 (1H, td, *J* = 7.7, 2.0 Hz), and 8.53 (1H, dq, *J* = 4.7, 0.8 Hz); LC/MS (ESI) *m*/*z* 299 (M+H)^+^; HRMS (ESI) calcd for C_19_H_27_N_2_O (M+H)^+^ 299.2123, found 299.2116; HPLC *t*_R_ 3.6 min (99%).

#### 4.4.24 *N*-(2-(Pyridin-2-yl)ethyl)adamant-ane-1-carboxamide (**31**)

A white solid was obtained in 88% yield. mp 76–79 °C; ^1^H NMR (CDCl_3_) *δ* 1.62–1.72 (6H, m), 1.78 (6H, d, *J* = 2.6 Hz), 2.00 (3H, br s), 2.98 (2H, t, *J* = 6.4 Hz), 3.60 (2H, q, *J* = 6.4 Hz), 6.70 (1H, br, NH), 7.10–7.18 (2H, m), 7.62 (1H, td, *J* = 7.9, 1.7 Hz) and 8.52 (1H, d, *J* = 4.4 Hz). LC/MS (ESI) *m*/*z* 285 (M+H)^+^; HRMS (ESI) calcd for C_18_H_25_N_2_O (M+H)^+^ 285.1967, found: 285.1977; HPLC *t*_R_ 2.5 min (99%).

#### 4.4.25 *N*-(2-(Pyridin-3-yl)ethyl)adamant-ane-1-carboxamide (**32**)

A white solid was obtained in 72% yield. mp 83–85 °C; ^1^H NMR (CDCl_3_) *δ* 1.02–1.12 (12H, m), 1.34 (3H, br s), 2.16 (2H, t, *J* = 6.2 Hz), 2.82 (2H, q, *J* = 6.2 Hz), 4.95 (1H, br, NH), 6.58 (1H, m), 6.85 (1H, m) and 7.82 (2H, m); LC/MS (ESI) *m*/*z* 283 (M−H)^+^; HRMS (ESI) calcd for C_18_H_25_N_2_O (M+H)^+^ 285.1967, found: 285.1968; HPLC *t*_R_ 2.5 min (99%).

#### 4.4.26 2-(Adamantan-1-yl)-*N*-methyl-*N*-(thiophen-2-ylmethyl)acetamide (**4**)

A white solid was obtained in 68% yield. mp 107–109 °C; ^1^H NMR (CDCl_3_) *δ* 1.62–1.77 (12H, m), 2.00 (3H, br s), 2.19 (2H, s), 3.00 (3H, s), 4.52 (2H, s), 6.95 (2H, m) and 7.23 (1H, dd, *J* = 5.0, 1.3 Hz); LC/MS (ESI) *m*/*z* 304 (M+H)^+^; HRMS (ESI) calcd for C_18_H_26_NOS (M+H)^+^ 304.1735, found 304.1726; HPLC *t*_R_ 3.8 min (99%).

#### 4.4.27 2-(Adamantan-1-yl)-*N*-(thiophen-2-ylmeth-yl)acetamide (**33**)

A white solid was obtained in 62% yield. mp 117–119 °C; ^1^H NMR (CDCl_3_) *δ* 1.56–1.71 (12H, m), 1.93 (2H, s), 1.95 (3H, br s), 4.60 (2H, d, *J* = 5.2 Hz), 5.63 (1H, br, NH), 6.90–6.95 (2H, m) and 7.21 (1H, dd, *J* = 4.7, 1.5 Hz); LC/MS (ESI) *m*/*z* 288 (M−H)^+^; HRMS (ESI) calcd for C_17_H_24_NOS (M+H)^+^ 290.1579, found 290.1585; HPLC *t*_R_ 2.8 min (99%).

#### 4.4.28 2-(Adamantan-1-yl)-*N*-methyl-*N*-(thio-phen-3-ylmethyl)acetamide (**34**)

A white solid was obtained in 62% yield. mp 117–119 °C; ^1^H NMR (CDCl_3_) signals from rotamers in 2:1 ratio: *δ* 1.55–1.75 (12H, m), 1.95 (3H, br), 2.16 (2H, s), 2.95 (3H, s), 4.57 (2H, s), 7.02 (1H, m), 7.11 (1H, m) and 7.26 (1H, m); *δ* 1.55–1.75 (12H, m), 1.95 (3H, br), 2.19 (2H, s), 2.92 (3H, s), 4.54 (2H, s), 6.91 (1H, m), 7.02 (1H, m) and 7.33 (1H, m); LC/MS (ESI) *m*/*z* 304 (M+H)^+^; HRMS (ESI) calcd for C_18_H_26_NOS (M+H)^+^ 304.1735, found 304.1718; HPLC *t*_R_ 2.9 min (99%).

#### 4.4.29 2-(Adamantan-1-yl)-*N*-methyl-*N*-[(1-methyl-1*H*-pyrrol-2-yl)methyl]acetamide (**35**)

A white solid was obtained in 50% yield. mp 75–76.5 °C; ^1^H NMR (CDCl_3_) *δ* 1.60–1.72 (12H, m), 1.95 (3H, br), 2.14 (2H, s), 2.88 (3H, s), 3.57 (3H, s), 4.59 (2H, s), 6.03–6.07 (2H, m) and 6.59 (1H, t, *J* = 2.1 Hz); LC/MS (ESI) *m*/*z* 323 (M+Na)^+^; HRMS (ESI) calcd for C_19_H_29_N_2_O (M+H)^+^ 301.2280, found: 301.2272; HPLC *t*_R_ 2.8 min (98%).

#### 4.4.30 2-(Adamantan-1-yl)-*N*-[(1-methyl-1*H*-pyrrol-2-yl)methyl]acetamide (**36**)

A white solid was obtained in 50% yield. mp 92–95 °C; ^1^H NMR (CDCl_3_) *δ* 1.55 (6H, d, *J* = 2.8 Hz), 1.57–1.62 (6H, m), 1.85 (2H, s), 1.91 (3H, br), 3.51 (3H, s), 4.35 (2H, d, *J* = 5.2 Hz), 5.30 (1H, br, NH), 6.00(2H, d, *J* = 2.3 Hz) and 6.55 (1H, t, *J* = 2.2 Hz); LC/MS (ESI) *m*/*z* 285 (M−H)^+^; HRMS (ESI) calcd for C_18_H_27_N_2_O (M+H)^+^ 287.2123, found: 287.2112; HPLC *t*_R_ 2.9 min (99%).

#### 4.4.31 2-(Adamantan-1-yl)-*N*-methyl-*N*-(pyridin-3-ylmethyl)acetamide (**37**)

An off-white solid was obtained in 52% yield. mp 67–68.5 °C; ^1^H NMR (CDCl_3_) *δ* 1.58–1.72 (12H, m), 1.96 (3H, br), 2.19 (2H, s), 2.97 (3H,), 4.60 (2H, s), 7.24 (1H, m), 7.65 (1H, dt, *J* = 8.2, 1.7 Hz) and 8.47-8.57 (2H, m); LC/MS (ESI) *m*/*z* 299 (M+H)^+^; HRMS (ESI) calcd for C_19_H_27_N_2_O (M+H)^+^ 299.2123, found: 299.2130; HPLC *t*_R_ 2.1 min (98%).

#### 4.4.32 2-(Adamantan-1-yl)-*N*-(pyridin-2-yl-methyl)acetamide (**38**)

A white solid was obtained in 86% yield. mp 87–88.5 °C; ^1^H NMR (CDCl_3_) *δ* 1.58–1.70 (12H, m), 1.95 (3H, br s), 2.02 (2H, s), 4.54 (2H, d, *J* = 4.9 Hz), 6.60 (1H, br, NH), 7.16–7.29 (2H, m), 7.65 (1H, td, *J* = 7.7, 2.0 Hz) and 8.52 (1H, d, *J* = 5.0 Hz); LC/MS (ESI) *m*/*z* 285 (M+H)^+^; HRMS (ESI) calcd for C_18_H_25_N_2_O (M+H)^+^ 285.1967, found: 285.1962; HPLC *t*_R_ 2.5 min (99%).

#### 4.4.33 *N*-Methyl-*N*-(thiophen-2-ylmethyl)-*t*_R_icyclo[3.3.1.0^(3,7)^]nonane-3- carboxamide (**39**)

A colourless oil was obtained in 86% yield. mp 87–88.5 °C; ^1^H NMR (CDCl_3_) *δ* 1.59–1.68 (4H, m), 1.87–1.95 (4H, m), 2.10–2.15 (2H, m), 2.32 (2H, br s), 2.91 (1H, bt, *J* = 6.6 Hz), 3.02 (3H, br s, NCH_3_), 4.74 (2H, s), 6.95 (2H, m, *J* = 3.4 Hz) and 7.21–7.25 (1H, m); LC/MS (ESI) *m*/*z* 276 (M+H)^+^; HRMS (ESI) calcd for C_16_H_22_NOS (M+H)^+^ 276.1422, found: 276.1425; HPLC *t*_R_ 2.8 min (97%).

#### 4.4.34 *N*,1-Dimethyl-*N*-(thiophen-2-ylmethyl)cyclohexanecarboxamide (**40**)

A clear oil was obtained in 83% yield. ^1^H NMR (CDCl_3_) *δ* 1.25 (3H, s), 1.31–1.41 (3H, m), 1.47–1.58 (5H, m), 2.10–2.16 (2H, m), 3.06 (3H, s), 4.73 (2H, s), 6.94–6.96 (2H, m), 7.22–7.24 (1H, m); LC/MS (ESI) *m*/*z* 252 (M+H)^+^; HRMS (ESI) calcd for C_14_H_22_NOS (M+H)^+^ 252.1422, found: 252.1421; HPLC *t*_R_ 2.7 min (99%).

#### 4.4.35 *N*-Methyl-*N*-(thiophen-2-ylmethyl)-1-p-tolylcyclopropanecarboxamide (**41**)

A clear oil was obtained in 53% yield. ^1^H NMR (CDCl_3_) *δ* 1.15 (2H, br s), 1.41 (2H, br s), 2.29 (3H, s), 2.80 (3H, s), 4.70 (2H, s), 6.91 (1H, s) and 7.05–7.26 (6H, m); LC/MS (ESI) *m*/*z* 308 (M+Na)^+^; HRMS (ESI) calcd for C_17_H_20_NOS (M+H)^+^ 286.1266, found 286.1246; HPLC *t*_R_ 2.3 min (99%).

#### 4.4.36 1-(4-Chlorophenyl)-*N*-methyl-*N*-(thio-phen-2-ylmethyl) cyclopropanecarbox-amide (**42**)

A clear oil was obtained in 72% yield. ^1^H NMR (CDCl_3_) *δ* 1.15 (2H, br s), 1.45 (2H, br s), 2.79 (3H, s), 4.70 (2H, s), 7.02 (1H, s), 7.07 (2H, br s), and 7.21–7.24 (4H, m); LC/MS (ESI) *m*/*z* 328 (M+Na)^+^; HRMS (ESI) calcd for C_16_H_17_ClNOS (M+H)^+^ 306.0719, found: 306.0707; HPLC *t*_R_ 2.3 min (99%).

#### 4.4.37 1-(4-Chlorophenyl)-*N*-methyl-*N*-(thio-phen-2-ylmethyl)cyclobutanecarbox-amide (**43**)

A clear oil was obtained in 49% yield. ^1^H NMR (CDCl_3_) *δ* 1.95 (2H, m), 2.34 (2H, t, *J* = 9.5 Hz), 2.47 (3H, s), 2.86 (2H, t, *J* = 10 Hz), 4.68 (2H, s), 6.89 (2H, br s) and 7.20–7.31 (5H, m); LC/MS (ESI) *m*/*z* 342 (M+Na)^+^; HRMS (ESI) calcd for C_17_H_19_ClNOS (M+H)^+^ 320.0876, found: 320.0861; HPLC *t*_R_ 2.7 min (99%).

#### 4.4.38 1-(4-Chlorophenyl)-*N*-methyl-*N*-(thio-phen-2-ylmethyl)cyclopentanecarbox-amide (**44**)

A white solid was obtained in 88% yield. mp 58–60 °C; ^1^H NMR (CDCl_3_) *δ* 1.62–1.82 (4H, m), 1.93–2.10 (3H, m), 2.43–2.52 (1H, m), 2.53 (3H, br s), 4.69 (2H, br s), 6.92 (2H, d, *J* = 3.0 Hz), 7.12-7.24 (5H, m); LC/MS (ESI) *m*/*z* 334 (M+H)^+^; HRMS (ESI) calcd for C_18_H_21_ClNOS (M+H)^+^ 334.1032, found: 334.1029; HPLC *t*_R_ 3.1 min (99%).

## Figures and Tables

**Table 1:** Human 11β-HSD1 inhibition by adamantyl carboxamide derivatives **3**, **5**–**19**

**Figure:**
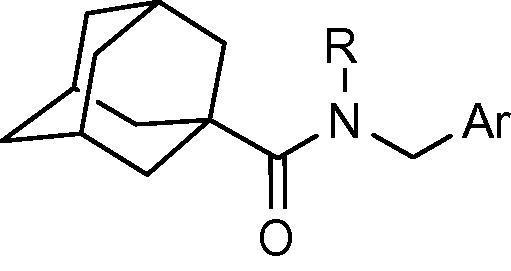

Compd.	R	Ar	IC_50_ (nM)
**3**	Me		229
**5**	H		403
**6**	Me		275
**7**	Me		12%^a^
**8**	Me		55%^a^
**9**	H		8%^a^
**10**	Me		32%^a^
**11**	Me		29%^a^
**12**	Me		125
**13**	Me		249
**14**	H		68%^a^
**15**	Me		114
**16**	H		1700
**17**	Me		24%^a^
**18**	Me		34%^a^
**19**	Me		31%^a^

‘^∗^Indicates point of attachment.’

a Percentage of inhibition measured at 1 μM.

**Table 2:** Human 11β-HSD1 inhibition by adamantyl carboxamide derivatives **20**–**27**

**Figure:**
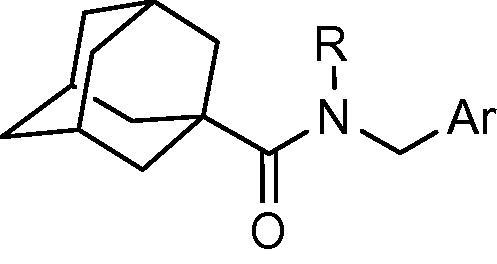

Compd.	R	Ar	IC_50_ (nM)
**20**	Me		118
**21**	Me		22%^a^
**22**	Me		23%^a^
**23**	Me		17%^a^
**24**	Me		57%^a^
**25**	H		676
**26**	H		45%^a^
**27**	Me		48%^a^

‘^∗^Indicates point of attachment.’

a Percentage of inhibition measured at 1 μM.

**Table 3:** Human 11β-HSD1 inhibition by adamantyl carboxamide derivatives **28**–**32**

**Figure:**
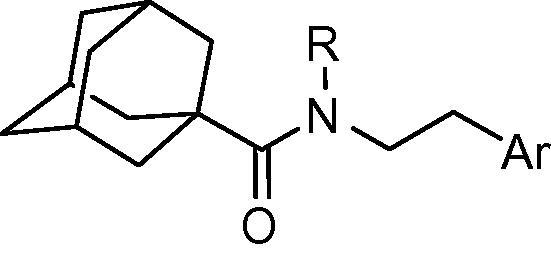

Compd.	R	Ar	IC_50_ (nM)
**28**	Me		448
**29**	H		679
**30**	Me		165
**31**	H		379
**32**	H		1%^a^

‘∗Indicates point of attachment.’

a Percentage of inhibition measured at 1 μM.

**Table 4:** Human 11β-HSD1 inhibition by adamantyl acetamide derivatives **4, 33**–**38**

**Figure:**
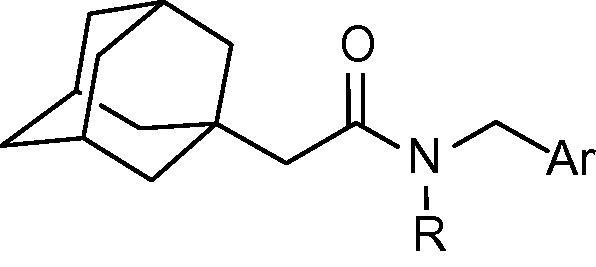

Compd.	R	Ar	IC_50_ (nM)
**4**	Me		308
**33**	H		66%^a^
**34**	Me		349
**35**	Me		655
**36**	H		2880
**37**	Me		67%^a^
**38**	Me		26%^a^

‘^∗^Indicates point of attachment.’

a Percentage of inhibition measured at 1 μM.

**Table 5:** Human 11β-HSD1 inhibition by non-adamantyl compounds 39–44

**Figure:**
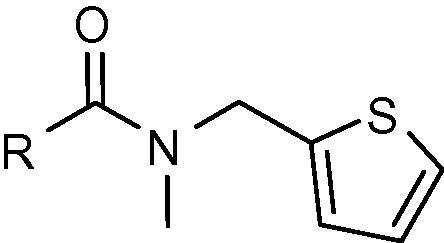

Compd.	R	IC_50_ (nM)
39		1080
40		3600
41		280
42		1550
43		385
44		2200

‘^∗^Indicates point of attachment.’

**Table 6:** Inhibition of human cytochrome P450 enzymes

CYP450	Compound			
	**3**	**4**	**15**^b^	**41**^b^
1A2	6%^a^	1%^a^	>100 μM	>100 μM
2C9	62%^a^	49%^a^	>100 μM	4.5 μM
2C19	13%^a^	80%^a^	>100 μM	>100 μM
2D6	2%^a^	9%^a^	>100 μM	72 μM
3A4-BFC	10 μM^b^	0.15 μM^b^	19 μM	7.3 μM
3A4-BQ	34%^a^	66%^a^	18 μM	1.6 μM

a Percentage inhibition measured at 10 μM.

b IC_50_ reported as the mean value of two measurements.
